# Effect of Selenium on Incidence and Severity of Mucositis during Radiotherapy in Patients with Head and Neck Cancer

**DOI:** 10.3290/j.ohpd.a45080

**Published:** 2020-09-04

**Authors:** Elahe Laali, Soheila Manifar, Ali Kazemian, Zahra Jahangard-Rafsanjani, Kheirollah Gholami

**Affiliations:** a Department of Clinical Pharmacy, Faculty of Pharmacy, Tehran University of Medical Sciences, Tehran, Iran. Experimental design, performed the experiments in partial fulfilment of requirements for a degree, evaluate patients during study, performed a certain test, consulted on and performed statistical evaluation, wrote and proofread the manuscript, contributed substantially to discussion.; b Assistant Professor, Assistant Professor, Department of Oral and Maxilla Facial Medicine, School of Dentistry, Imam khomeini Hospital Complex, Tehran, Iran Performed the experiments in partial fulfilment of requirements for a degree, evaluate patients during study.; c Associate Professor, Radiation Oncology Research Center (RORC), Cancer Institute, Tehran University of Medical Sciences, Tehran, Iran Experimental design, performed the experiments in partial fulfilment of requirements for a degree.; d Clinical Pharmacist, Associate Professor, Clinical Pharmacist, Department of Clinical Pharmacy, Faculty of Pharmacy, Tehran University of Medical Sciences, Tehran, Iran. Contribution to the paper: hypothesis, experimental design, proofread the manuscript, consulted on and performed statistical evaluation, contributed substantially to discussion.; e Professor, Research Center for Rational Use of Drugs, Tehran University of Medical Sciences, Tehran, Iran, Karim Khane Zand Avenue, Hafte-Tir Square, Tehran. Idea, hypothesis, contributed substantially to discussion.

**Keywords:** selenium, oral mucositis, head and neck cancer, radiation, concurrent chemotherapy, prevention

## Abstract

**Purpose:**

Oral mucositis (OM) is the most frequent side effect of radiation. Selenium deficiency leads to increased levels of free oxygen radicals and the selenium level tends to fall during radiotherapy. Hence, in this double-blind randomised controlled clinical trial, the effect of selenium was assessed in patients receiving radiation.

**Materials and Methods:**

Patients with head and neck cancer who were candidates to receive radiation were instructed to use selenium 200 mcg tablets twice daily. The grade of OM was evaluated by the World Health Organization (WHO) grading system on a weekly basis. The selenium level was measured at baseline and at the end of the radiation.

**Results:**

Seventy-one patients with head and neck cancer (37 in the selenium group, 34 in the placebo group) were enrolled in the study. The cumulative incidence of OM (grade 1–4) was 97.3% in the selenium and 100% in placebo group (p value: 0.79), and difference in the mean serum selenium level at the end of radiation was not statistically significant between the two groups (p value 0.24)

**Conclusion:**

Selenium supplementation does not appear to affect the selenium level as well as the severity and duration of OM. It is supposed that higher doses may be effective in the prevention of RT-mucositis. This trial was registered in the Iranian Registry of Clinical Trials accessible at www.irct.ir (ID No. IRCT2014072718612N1)

Head and neck cancer is the third most common cancer in the world. Treatment of head and neck cancer involves multidisciplinary strategies including surgery, radiotherapy (RT), chemotherapy, targeted therapy or a combination of the above.^13^ RT is one of the core treatment modalities in the head and neck cancer. While production of reactive oxygen species (ROS) increases the antitumor process through radiation,^4^ normal cells also experience dysfunction and damage.^26^ Oral mucositis (OM), a painful inflammation and ulceration of the mucous membranes, is the most frequent side effect of radiation with an incidence of 80–97%.^7,16,37^ OM develops in four phases. First, free radicals released by the mucosa cause inflammation. Second, epithelial turnover declines; erythema and atrophy start in this stage and microtraumas lead to ulceration. Third, pseudomembrane formation and microbial colonisation may cause infection. Finally, recovery and healing will be achieved in the fourth phase.^25^

In general, clinical features are diagnostic in OM. Indeed, several criteria are used for grading OM. The WHO has classified OM into five grades of anatomical change from no change (stage 0) to ulceration with necrosis (stage 4) based on objective and subjective variables of OM.^27^

Cumulative doses of radiation have a crucial effect on the severity and onset of OM. At cumulative doses of 10 Gy, erythema appears and patients feel a burning sensation. After about 2 weeks when the patient has received 30 Gy of radiation, unbearable complications such as pain and inability to chew and eat appear.^32,34^ Grade 3 and 4 (G3, G4), which are considered a severe form of OM, lead to unplanned interruption in the treatment, worsening the patient’s quality of life and survival.^3^ Therefore, prevention of severe OM is important in the cancer treatment process.

Studies have evaluated several preventive and symptom-managing agents.^7,14,29,30^ Although some of them have significantly improved the symptoms of radiation OM, none of them has been approved by caregivers and international guidelines.^19^

Since inflammation and ROS formation have important roles in OM pathogenesis, antioxidants and anti-inflammatory agents have been evaluated for prevention of OM in several studies with some benefits.^12,22^ Selenium, an essential trace element, plays an important role as a cofactor in glutathione peroxidase, an endogenous antioxidative system.^20^ Hence, selenium deficiency leads to increase levels of free oxygen radicals by diminishing the enzymatic activity of glutathione peroxidase and the selenium level tends to fall during radiotherapy.^8,21^ Additionally, adverse effects, interruption of cancer treatment and quality of life are related to the selenium level. Furthermore, studies have shown that selenium can reduce the grade and incidence of radiation and chemotherapy OM.^12,24,27,28^

Hence, in this double-blind randomised controlled clinical trial, the effect of selenium supplementation on the incidence and severity of OM was assessed in patients receiving radiotherapy, with or without chemotherapy.

## Materials and Methods

This double-blind placebo-controlled randomised clinical trial was conducted in Imam Khomeini Hospital Complex, Tehran, Iran, from February 2015 to March 2017. Patients with head and neck cancer who were scheduled to receive radiation as part of their cancer treatment were enrolled in this study. This trial was registered in the Iranian Registry of Clinical Trials available at www.irct.ir (ID No. IRCT2014072718612N1).

### Ethics

The study was approved by the Ethics Committee of Tehran University of Medical Sciences and written informed consent was obtained from all participants.

### Sample Size

The study sample size was calculated 84 patients (42 participants in each study group) assuming a 30% decrease in the incidence of OM according to the result of a pilot study, with a statistical power 80% and a two-sided signiﬁcance level of 5%.^5^

### Patient Selection

Patients with head and neck cancer who were due to receive radiation on at least two-thirds of the oral cavity were included in the study. Patients aged 18–85 years with a normal renal function (creatinine clearance > 60 ml/min) and an acceptable performance status (Karnofsky performance status > 70%) without any history of RT were eligible for this study. Patients were randomly divided into two treatment and control groups in a blocked randomisation schedule. The radiation protocol in all participants (either definitive or postoperative) was conventional RT techniques, 5 fractions per week at 60–70 Gy cumulative doses over 6 to 7 weeks with or without cisplatin (30–50 mg/m^2^ weekly, during radiation) as a radiosensitiser.

### Intervention

The participants were given a bottle containing 100 tablets of selenium (Webber Naturals, Coquitlam, BC, Canada, 200 mcg) or placebo. The subjects were instructed to use the tablets twice daily from the first day of radiation until the end of radiotherapy (including days without radiation exposure) to prevent OM as a side effect of radiation. Adherence to medicine consumption was checked by phone call on a weekly basis.

In addition to maintaining good oral hygiene, the participants were educated to use a saline rinse frequently.^17^ An oral cavity specialist visited the oral cavity in both groups to identify any dental problems before treatment started. The same treatment protocol was ordered for all patients who suffered from OM during treatment.

### OM Assessments

The grade of OM was evaluated by the WHO oral toxicity scale.^18^ The WHO scoring was done as follows: grade zero = normal, no OM; grade 1 = soreness and erythema; grade 2 = erythema, ulcers, can eat solids; grade 3 = ulcers, requires liquid diet only; grade 4 = alimentation not possible).^35^ The assessments were performed by an assigned oral medicine specialist (SM) and one author (EL) who were both blind to groups.

The oral status of each patient was evaluated on the first day and then on a weekly basis until radiotherapy finished, and then after one month. Objective or subjective symptoms were checked by these two criteria to determine the incidence, severity and duration of OM in the participants.

### Chemotherapy Subgroup

The grade of neutropenia and renal failure was evaluated based on the NCI CTCAE ver. 4.03. A creatinine level increase of > 0.3 mg/dL or 1.5–2.0 mg/dL above baseline was considered as renal failure and a neutrophil count decrease to 1000–500 mm^3^ was considered as grade 3 of this toxicity.

### Selenium Level Monitoring

Serum samples were obtained from both groups at baseline and at the end of radiotherapy. Selenium supplementation was discontinued at least 24 h before sampling. The serum samples were stored in two separate microtubes at –80°C until selenium assay was done. The selenium level was determined using the graphite furnace atomic absorption spectrometry (Atomic Absorption, Agilent, Germany, selenium level reported in mcg/L).

### Statistical Evaluation

The SPSS software was used to analyse the data and p values less than 0.05 were considered statistically significant.

## Results

**Fig 1:**
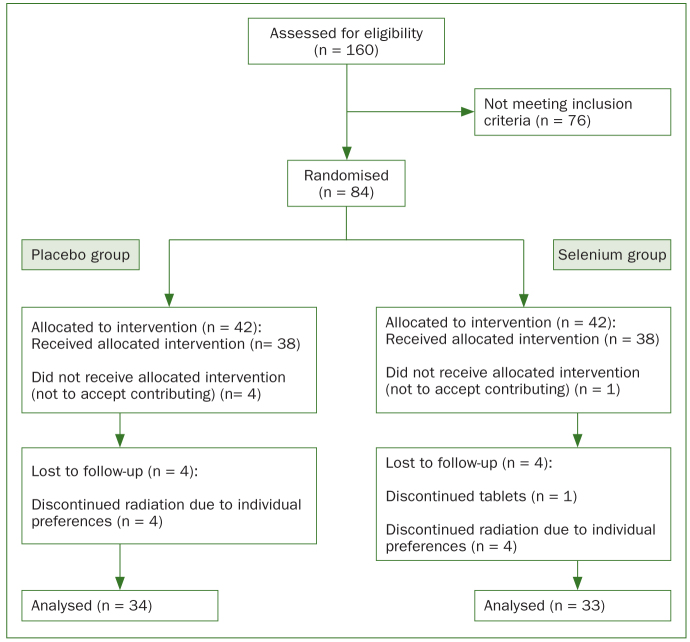
Study participants ﬂow diagram.

Initially 84 patients were recruited for this investigation, of whom 13 patients dropped out after initiation of radiotherapy. Consequently, there were 37 and 34 patients in the selenium and placebo group, respectively. These patients were followed-up and analysed (Fig 1). The baseline characteristics of the patients were similar in both study groups. The patients’ data are summarised in Table 1.

**Table 1:** Baseline characteristics of the patients

Characteristics	Selenium group n = 37	Placebo group n = 34
Sex n (%)		
Male	25 (67.9)	25 (73.5)
Age, year		
Mean Median Minimum–Maximum	52.14 51 22–81	54.74 57.50 18–81
Cancer type, n (%)		
SCC NPC ADC Melanoma Neuroblastoma Metastasis of other cancer	25 (67.6) 4 (10.8) 4 (10.8) 1 (2.4) 1 (2.4) 2 (5.4)	26 (76.5) 4 (11.8) 0 (0) 1 (2.9) 1 (2.9) 2 (5.9)
Total radiation dose, n (%)		
≤6000 Gy >6000 Gy	19 (51.3) 18 (48.6)	15 (44.1) 19 (55.9)
Concurrent chemotherapy, n (%)	18 (48.6)	16 (47.1)

SCC, Squamous cell carcinomas; NPC, nasopharyngeal carcinoma; ADC, adenocarcinoma.

**Fig 2:**
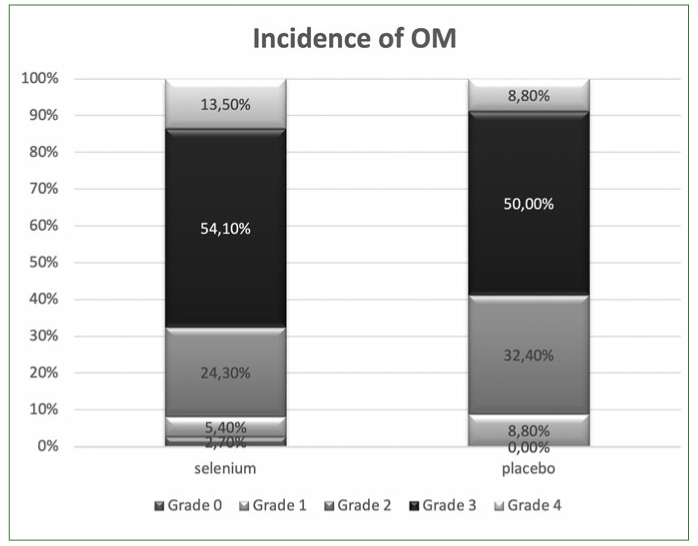
Cumulative incidence of the OM (grade 1–4).

Almost all patients experienced some degree of OM. The cumulative incidence of OM (grade 1–4) was not significantly different between the two groups (97.3% in selenium and 100% in the placebo group, p value: 0.79, Fig 2).

**Fig 3:**
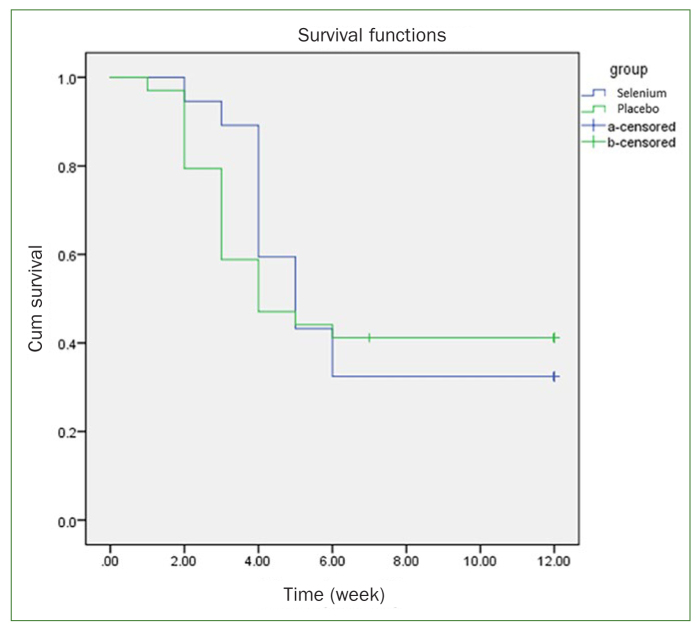
Kaplan–Meier curve, developing severe OM.

Severe OM (grade 3, 4) was seen in 25 and 20 patients in the selenium and placebo group, respectively. According to severe OM development, the log rank analysis showed no difference between the two groups (p value 0.78). However, the Kaplan-Meier graph (Fig 3) showed a difference in the severity of OM between the two groups in the third week (p value 0.78). Meanwhile, linear-by-linear association analysis showed a statistically significant difference in severe OM incidence between two groups in week 3 (p value 0.017; 42% in the placebo group and 9.8% in the selenium group).

The mean duration of OM (grade 1–4) was not different between the two groups (46.97 ± 20.26 days in the selenium and 50.44 ± 17.56 days in the placebo group, p value 0.27).

Other evaluated OM variables such as the onset and recovery time of OM and duration of severe OM are presented in Table 2.

**Table 2:** Effect of selenium in OM (Mean ± SD)

Variable	Selenium group	Placebo group	P value
Duration of OM (day) Grade 1–4	46.97 ± 20.26	50.44 ± 17.56	0.27
Duration of severe OM (day) Grade 3, 4	10.97 ± 11.49	14.00 ± 13.67	0.34
Onset of OM (week)	1.61 ± 0.83	1.70 ± 1.05	0.31
Recovery (day after radiation completion)	8.37 ± 11.39	8.88 ± 11.09	0.80

Evaluation of the concurrent chemoradiation subgroup (n = 18 in the selenium group and n = 16 in the placebo group) showed no statistically significant differences in the incidence of other adverse effects related to chemo-radiation such as neutropenia (2 patients in selenium and 3 patients in placebo group with grade 3 neutropenia) and renal failure (7 patients in selenium group compare to 3 patients in placebo group) between the two groups.

Regarding the serum selenium level, alterations of selenium level were not statistically significant different between the two groups (rise in the selenium level: 17.30 ± 36.48 mcg/L in the selenium and 7.15 ± 34.92 mcg/L in the placebo group, p value 0.50). Moreover, there was no statistically significant difference in the mean serum selenium level at the end of radiation between the two groups (p value 0.24). The data of the selenium level are shown in Table 3.

**Table 3:** Serum selenium level (mean ± SD (mcg/L)

Group	Selenium group	Placebo group	P value
Selenium level Before radiation	79.49 ± 25.63	85.38 ± 30.63	0.35
Selenium level After radiation	97.42 ± 29.21	93.32 ± 33.26	0.50
Selenium level alterations	17.30 ± 36.48	7.15 ± 34.92	0.24

Interestingly, based on the selenium level before radiation, developing severe OM was statistically significant postponed in patients who had selenium levels ≥ 65 mcg/L (p value 0.04).

## Discussion

Radiotherapy, as a modality of head and neck cancer treatment, causes many side effects such as severe OM, leading to radiation interruption, treatment cessation and reduced quality of life.^38^ Proinflammatory cytokines generated by DNA damage result in injuries and tissue destruction.^36^ There are many inconclusive studies on the prevention of OM in cancerous patients.^7,23^ However, oral care and non-pharmacological mouth washes such as normal saline and also supplementations like oral zinc and vitamin E are suggested in guidelines.^2,17,23^

In this study, we evaluated the prophylactic effect of selenium supplementation on the incidence and severity of OM. Our results demonstrated that administration of selenium had no statistically significant effect on the incidence, duration and severity of radiation OM. However, according to cell culture analyses, selenium acts as a normal cell radio-protectant and high concentrations of sodium selenite in endothelial cells decrease the effects of radiation and highlight the possible normal cell protective effect of selenium in radiation.^31,33^

According to this survey, there was no statistically significant rise in the plasma concentration of selenium during the study. Therefore, ineffectiveness of selenium in reducing radiation OM might be due to the insufficient selenium concentration in target cells. Hence, the evaluated dose of selenium in this study (400 mcg) may not have been sufficient to increase the level of selenium in patients receiving radiation. Lack of adherence may contribute to this finding because we checked the adherence by only self-report. These findings are similar to the results of a study by Kiremidjian that showed no statistically significant rise in the selenium level following the oral intake of 200 μg selenium in head and neck cancer patients.^15^ However, some studies have shown that supplementation with recommended dietary allowance of selenium (400 mcg) causes a statistically significant rise in other situations. Jahangard performed a randomised controlled trial (RCT) on patients who underwent allogeneic haematopoietic stem cell transplantation (HSCT) and showed that the incidence of severe OM decreased following an increase in the selenium level although selenium was administered for 14 days in that study. However, studies have shown that selenium can decrease other radiation side effects such as diarrhoea and dysphagia in the head and neck, cervical and uterine cancer.^6,12,24^

The role of the cumulative radiation dose in starting, progression and ulceration of OM was evaluated in a review study in 2012. The results showed that OM became severe after receiving 20 Gy, which was about week 3 of treatment.^32^ The results of our study showed that the onset of OM was similar in both groups. However, in week 3 of radiation, selenium could decrease incidence of severe OM. Therefore, it can be concluded that if the dose of selenium is increased concurrently with increasing the radiation cumulative dose, severe OM may be prevented.

According to our findings, lower levels of selenium had a negative impact on developing severe OM, as patients with selenium levels below 65 mcg/L before radiation developed OM earlier. It can be hypothesised that correction of the selenium level before starting treatment may be a key point.

Nephropathy and bone marrow suppression are common cisplatin toxicities in chemotherapy regimen and the protective role of selenium in the reduction of these side effects has been shown in some studies.^9,11^ Cisplatin, as a radiosensitiser, is administrated at lower doses compared to the chemotherapy regimen.^1,10^ In this study, renal failure and grade 3 neutropenia occurred in a small group of patients with no statistically significant difference between the two groups.

### Limitation

Oral selenium supplementation adherence might affect the outcome interpretation.

As a strength of this study, it was the first double-blind, randomised, controlled study to maximise its internal validity. Moreover, using an oral cavity specialist enhanced the accuracy of the results.

## Conclusion

In conclusion, selenium supplementation at a dose of 400 mcg per day during radiation has no effects on the selenium level or the incidence and severity of OM. We believe that higher doses or other routes of administration may be effective in the prevention of radiation OM.
